# Morphological Characteristics of Drug-Eluting Biodegradable Polymeric Thin Films Developed on the Surface of Intraocular Lenses by Three Techniques: A Comparative Study

**DOI:** 10.7759/cureus.19674

**Published:** 2021-11-17

**Authors:** Athanasios Karamitsos, Lampros Lamprogiannis, Varvara Karagkiozaki, Aphrodite Koutsogianni, Zena Chakim, Nikolaos G Ziakas, Ioannis Tsinopoulos, Stergios Logothetidis

**Affiliations:** 1 2nd Department of Ophthalmology, Papageorgiou General Hospital / Aristotle University of Thessaloniki, Thessaloniki, GRC; 2 Lab for Thin Films - Nanobiomaterials - Nanosystems & Nanometrology (LTFN), Aristotle University of Thessaloniki, Thessaloniki, GRC; 3 Department of Ophthalmology, North Middlesex University Hospital, London, GBR

**Keywords:** dexamethasone, cataract, thin films, ocular drug delivery, nanotechnology, intraocular lens

## Abstract

Background

Cataract surgery is a very popular operation that requires a postoperative period of frequent instillation of antibiotic and anti-inflammatory eye drops. Modified drug-eluting intraocular lenses (IOLs) may eliminate the need for eye drops after surgery.

Aim

The purpose of this study is to compare the morphological characteristics of dexamethasone eluting biodegradable polymeric thin films developed on the surface of commercially available IOLs by three different methods.

Method

This experimental study was conducted between May and August of 2021 in the Lab for Thin Films - Nanobiomaterials - Nanosystems & Nanometrology (LTFN) of the Aristotle University of Thessaloniki. A mixture of two organic polymers [Poly (D, L-lactide-co-glycolide)(PLGA), lactide: glycolide (75:25) and Polycaprolactone (PCL)] and dexamethasone was prepared and then deposited on the surface of three-piece IOLs by spin coating, by spray coating, and by gravure printing. The modified IOLs were sterilized with the use of ultraviolet (UV) radiation and plasma treatment. Their structural properties were studied with the use of atomic force microscopy (AFM).

Results

Spin coating and gravure printing produced uniform thin films on the surface of the IOLs which were not damaged during the sterilization process. Spray coating led to the partial coating of the surface of the IOLs; the thin films underwent alterations following plasma treatment.

Conclusions

Thin films developed by spin coating and gravure printing on IOLs demonstrate the desired morphological characteristics that make them suitable candidates for further research.

## Introduction

A cataract is defined as the clouding of the crystalline lens. It is treated surgically, usually by removal of the cataractous lens and insertion of an artificial intraocular lens (IOL). This procedure is known as phacoemulsification with IOL insertion and is one of the most common surgical procedures worldwide [1]. Post-operatively, antibiotic and anti-inflammatory eye drops are instilled in the operated eye to prevent infection and to reduce inflammation [2]. This post-operative treatment commonly lasts between four and six weeks. As adherence to the eye drop regime is not optimal, alternative methods of delivery of the necessary therapeutics to the eye are being investigated [3]. 

Nanobiotechnology provides useful techniques for targeted and sustained drug delivery [4-6] and is, therefore, on the epicentre of the current research which aims to eliminate the need for eye drops after eye surgery. Different drug delivery systems have been suggested as viable alternatives to eye drops [7-10]. IOLs with appropriate modifications have been used as intraocular drug delivery systems [11-13]. Polymeric, dexamethasone-eluting, biodegradable thin films have previously been developed by the researchers on the surface of commercially available IOLs by spin coating with promising results [14-15]. This is a part of an ongoing project which intends to address the non-compliance with eye drops, by designing and fabricating an IOL-based system suitable for production at the industrial level.

Aiming to optimize the characteristics of the novel drug delivery system, the research team has proceeded to explore the suitability of other methods of coating IOLs with thin films. Spray coating and gravure printing have been used successfully in the past to produce biocompatible thin films in nanobiotechnology applications [16-17]. This research paper presents the comparison of the surface characteristics of lenses that have been modified by spin coating, spray coating, and gravure printing and the effects of the sterilization process on the polymeric coatings.

## Materials and methods

Materials

Two organic polymers were used:

1. Poly (D, L-lactide-co-glycolide), lactide: glycolide (75:25), Mw 66,000-107,000 [P1941, Sigma-Aldrich] [PLGA 75:25]

2. Polycaprolactone average Mw 48,000-90,000 [704105, Sigma-Aldrich] [PCL]

Previous studies have shown that these polymers are biocompatible and biodegradable, and they form thin films with pores, where the medicament can be encapsulated [18].

Dexamethasone (Dexamethasone powder ≥ 97 % [D4902], Sigma-Aldrich) was the anti-inflammatory therapeutic of choice; it is widely used in cataract surgery as part of the post-operative treatment, and it is known to be effective in limiting inflammation.

Commercially available three-piece acrylic intraocular lenses (MA60AC, Alcon Laboratories, USA) were used as substrates for the development of the thin films.

Development of the thin films on the surface of the IOLs

A solution consisting of a polymeric blend (PLGA 75:25 and PCL at a ratio of 9:1) and dexamethasone at a ratio of 3:1 in chloroform was prepared and then stirred for 12 hours with the use of a magnetic stirrer at room temperature. The concentration of the solution was 10 mg/ml.

A single layer thin film was developed on the surface of 21 IOLs. The modified lenses were allocated to three groups: Group A (Specimens 1-7), which included lenses that were modified by spin coating, Group B (Specimens 8-14), which included lenses that were modified by spray coating and Group C (Specimens 15-21), which included lenses that were modified by gravure printing.

Spin Coating

Group A lenses were fixated on glass and placed in the spin coating device within a nitrogen atmosphere glove box. The surface of the lenses was covered with 0.1 ml of the polymer-dexamethasone solution. An Eppendorf research ® adjustable pipette was used to ensure the uniform distribution of the solution.

The total duration of the spin coating was 36s. During the first 6s, the acceleration rate was 300 rpm/s, and this led to a speed of 650 rpm in 2.16 s. Subsequently, they spun for 30s and the acceleration rate of 500 rpm/s led to a speed of 2000 rpm within 2.6s. Deceleration was 500 rpm/s. The modified IOLs were left to slow dry for 24 hours.

Spray Coating

Group B lenses were also fixated on a glass plate using an adhesive material. The process was carried out in a nitrogen atmosphere glove box under constant conditions. 100μl of the solution were used. IOLs were sprayed at a constant rate from a height of 15 cm.

Gravure Printing

Group C lenses were fixated on the surface of a sheet-to-sheet (S2S) lab-scale gravure printing proofer via a PET film. Roll speed was 3 meters/minute.

Study of the morphology of the modified lenses

Atomic force microscopy (AFM) was used to study the morphology of the modified lenses. This is an established technique that can provide information regarding the surface of polymeric thin films, without damaging them. The study was performed under ambient environmental conditions; a scanning probe microscope (AFM Solver P-47H, NT-MDT, Moscow, Russia) was used to obtain detailed images of the novel drug delivery systems.

Sterilization

Two samples of each of the three groups underwent sterilization. One sample of each group was treated with plasma. A low-temperature plasma sterilizer (STERLINK® Advanced) was used, and the samples were treated for 15 minutes. The other sample was sterilized with the use of UV radiation. The modified IOLs were exposed to a UV-C lamp (253.7 nm, 250 mW/cm2 at 1 cm) for 20 minutes.

## Results

Morphological study of the drug delivery systems

**Figure 1:**
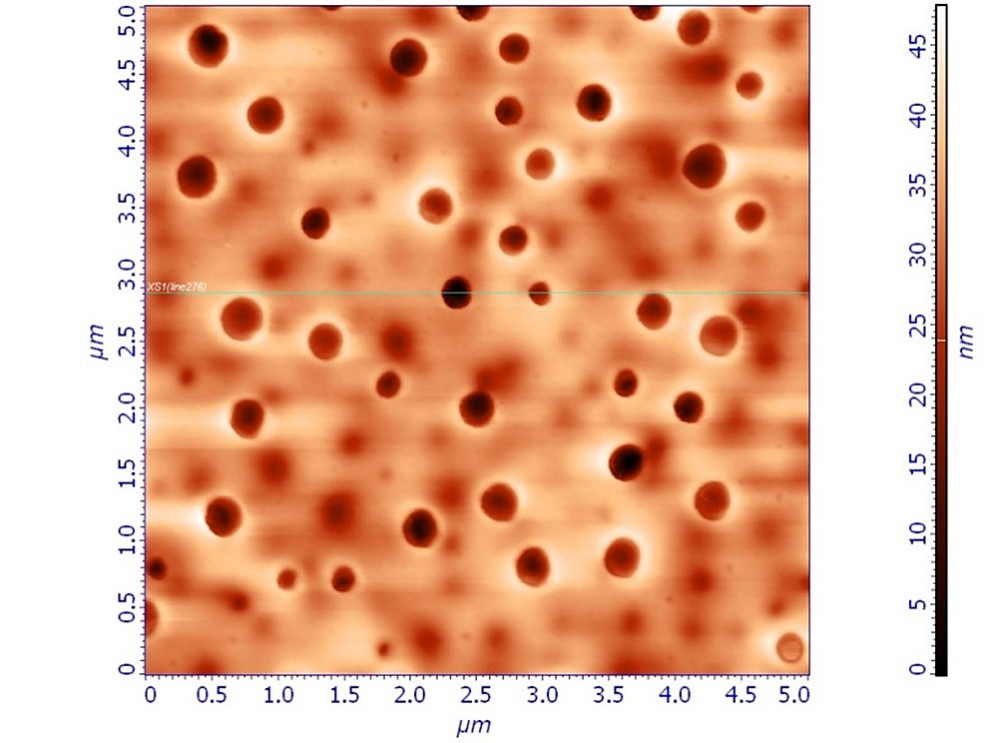
Topographical image of spin-coated thin film (Group A), 5 x 5 μm. Nanopores are depicted as round, darker areas.

**Figure 2:**
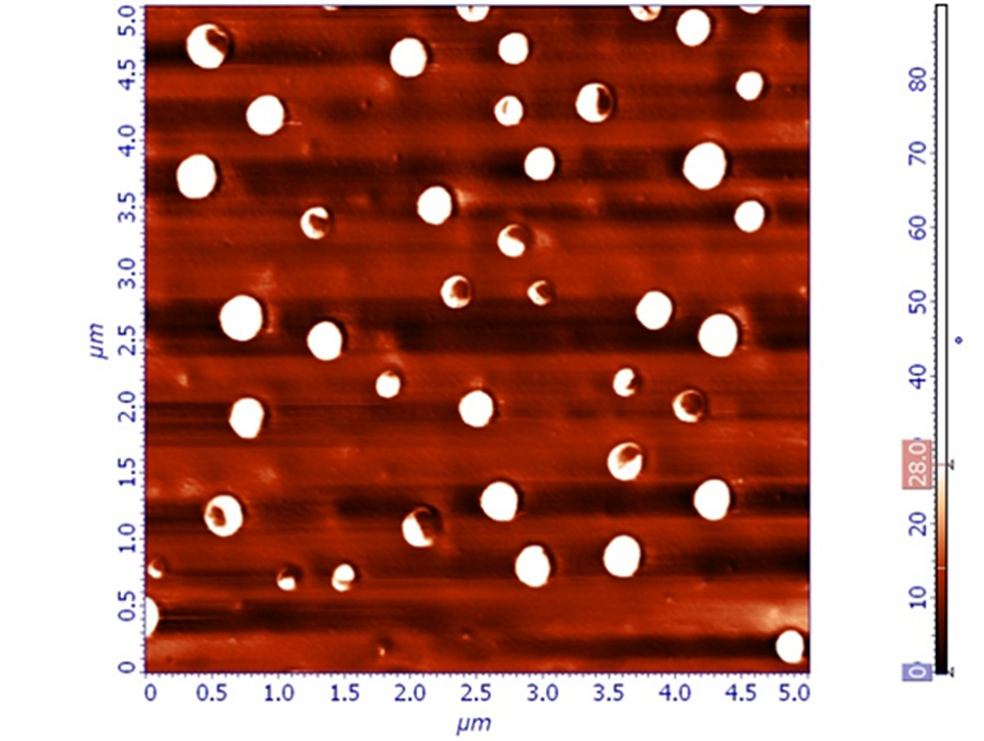
Phase image of spin-coated thin film (Group A), 5 x 5 μm. In phase images, nanopores are lighty coloured and dexamethasone can be observed as darker areas within the nanopores.

Samples of all three groups were studied with AFM and detailed images (topographic and phase images). Significant differences were noted between the three groups. Group A samples demonstrated a uniform porous single-layer thin film on the surface of the lens (Figure 1,2).

**Figure 3:**
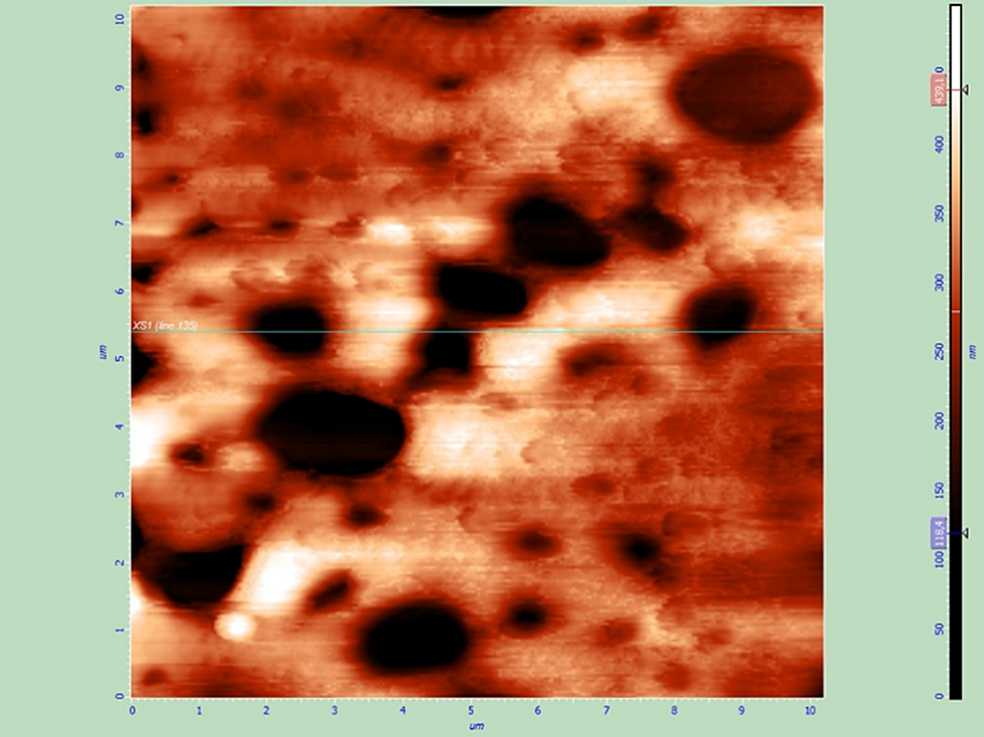
Topographical image of spray-coated thin film (Group B), 10 x 10 μm. Larger, non-symmetrical pores can be observed. The surface of the thin film appears less uniform, compared to the samples of the other groups

**Figure 4:**
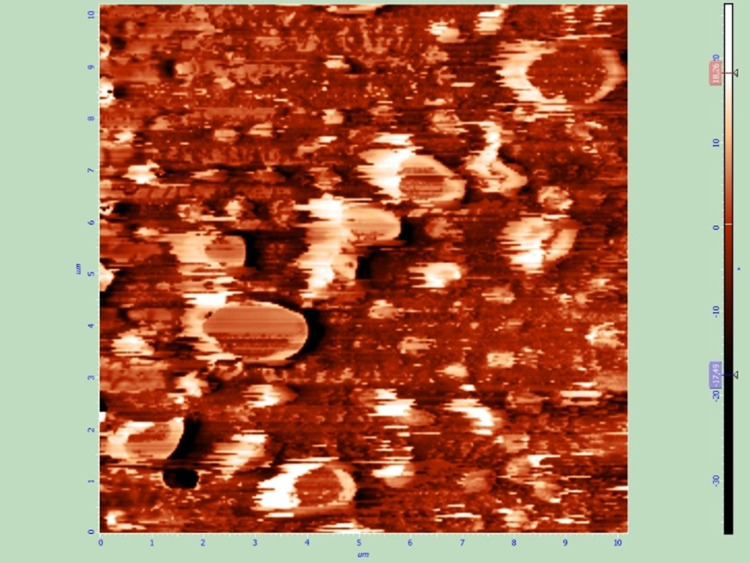
Phase image of spray-coated thin film (Group B), 10 x 10 μm. This phase image confirms the increased roughness of the coating.

Thin films produced by spray coating (Group B) did not cover all the surface of the lens. Superficial roughness was significantly higher than expected (Figure 3,4).

**Figure 5:**
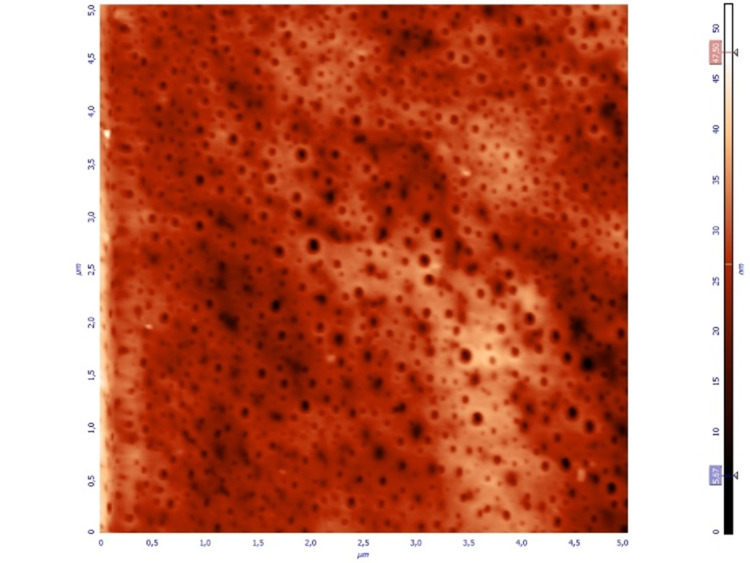
Topographical image of gravure printed thin film (Group C), 5 x 5 μm

**Figure 6:**
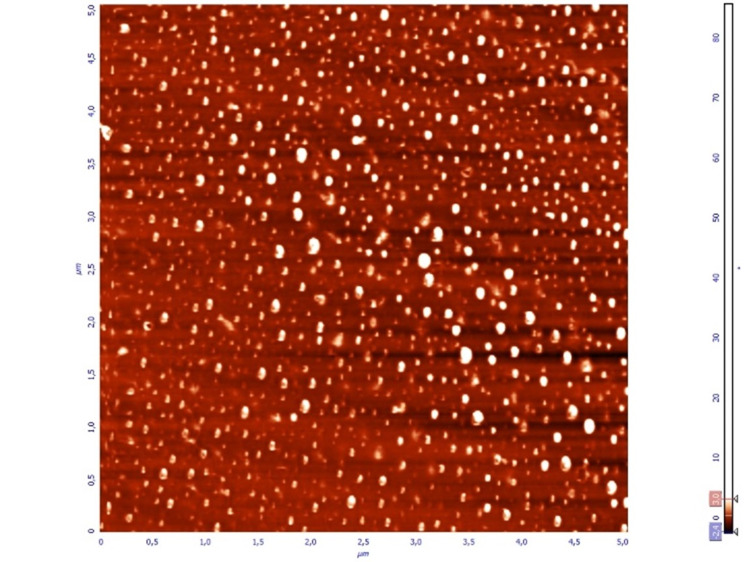
Phase image of gravure printed thin film (Group C), 5 x 5 μm

Gravure printed thin films (Group C) were uniform and covered the surface of the optic part of the lens without gaps, aggregates of dexamethasone or areas of increased roughness (Figure 5,6)

Sterilization and its effect on the thin films

**Figure 7:**
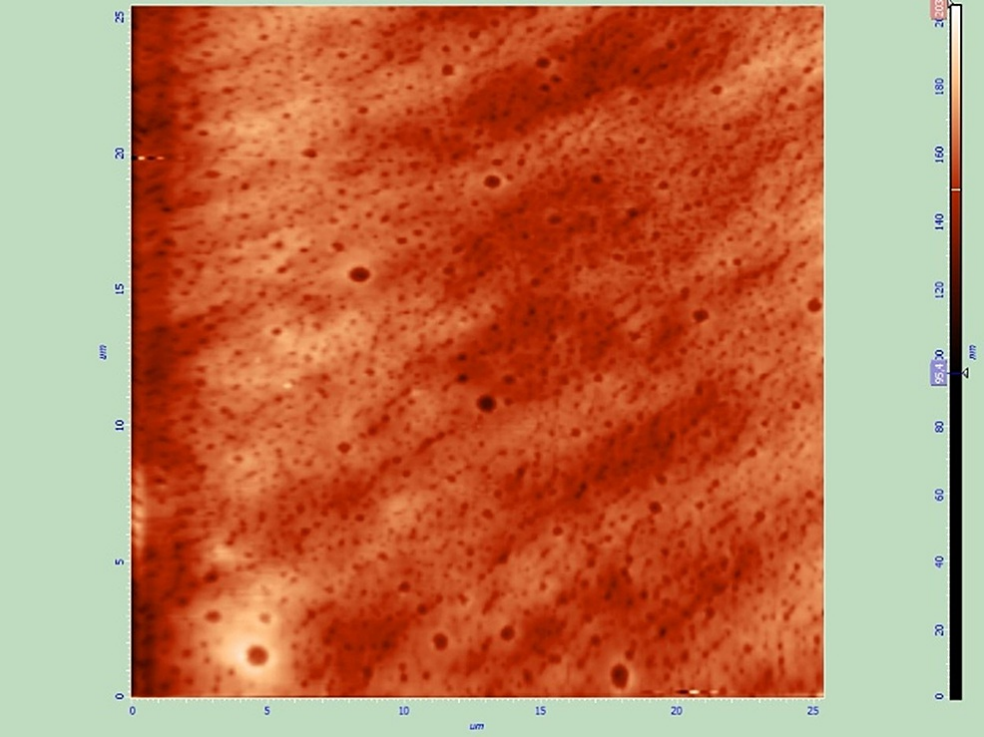
Spin coated thin film after sterilization with UV radiation, AFM topography image.

**Figure 8:**
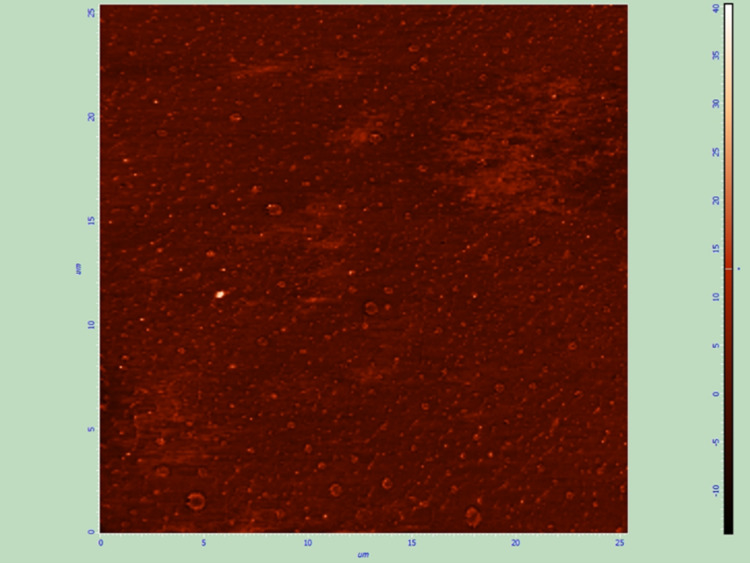
Spin coated thin film after sterilization with UV radiation, AFM phase image.

**Figure 9:**
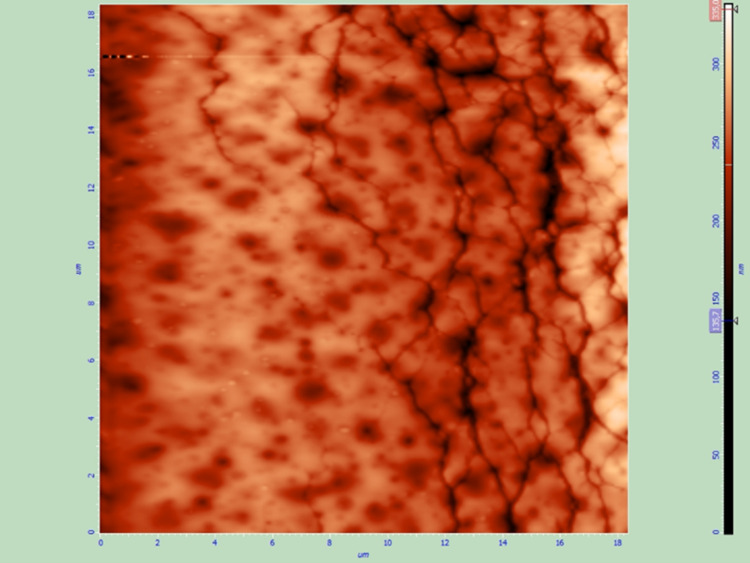
Spin coated thin film after sterilization with plasma treatment, AFM topography image. Non-preexisting cracks are visible on the thin film surface.

**Figure 10:**
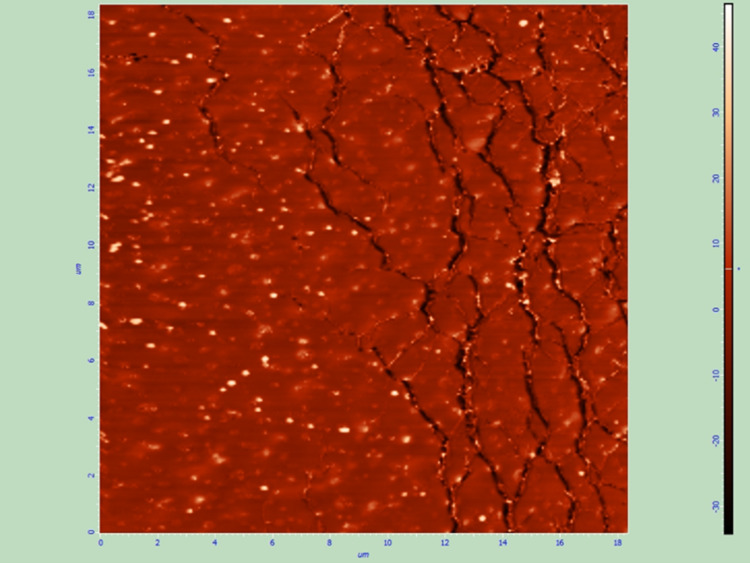
Spin coated thin film after sterilization with plasma treatment, AFM phase image.

All six samples (two of each of the three groups) were also examined by AFM to assess the effect of the two sterilization techniques on the thin films and the surface of the IOLs. All samples that were sterilized with UV radiation were not altered, while plasma treatment caused remarkable changes to the thin films such as extensive cracks (Figures 7-10).

## Discussion

Improvement of cataract surgical outcomes and patient satisfaction remains a priority for ophthalmic surgeons and researchers worldwide [19]. Eliminating the need for eye drops after the operation without compromising the safety and the comfort of the patient would certainly prove beneficial; all obstacles in the administration of eyedrops (intolerance, noncompliance, weak memory, difficulty with instillation) would be removed [20]. It is reasonable to expect that various approaches to dropless cataract surgery will gain interest in the foreseeable future [21]. Surodex© and DEXYCU© are two examples of commercially available biodegradable dexamethasone-releasing anterior chamber implants; dexamethasone is widely applied as the anti-inflammatory therapeutic agent of choice in intraocular drug delivery applications. [22-23]

This stage of our ongoing project aims to identify the most suitable method for thin film fabrication on the surface of IOLs. Spin coating, spray coating, and gravure printing have all been successfully used in similar biomedical applications. Assessment of the morphology of the thin films with AFM has indicated that spray coating was not as efficient as the other two techniques in coating the lenses’ surface with a uniform thin film. It can be argued that spray coating is a versatile technique and changing some of the parameters of the study (heat, spray distance) may lead to better outcomes. Spin coating has consistently produced good results, as previous studies have shown [14-15]. The optic part of the lenses that were modified by spin coating was fully coated by a polymeric monolayer with desired structural characteristics (uniform thickness, nanopores that serve as reservoirs for dexamethasone and contribute to its prolonged release). However, gravure printing is generally considered to be more industry-oriented, as it allows for simultaneous modification of more than one lenses [24]. Roll-to-roll techniques are established manufacturing technology platforms and are, as a result, more attractive in the context of industrial-scale production [25]. As the gravure-printed thin films have demonstrated satisfactory structural characteristics, it is reasonable that gravure printing may be the method of choice during further stages of this project.

Sterilization of the intraocular drug delivery systems is necessary to avoid infection after insertion of the modified lenses in the eye [26]. UV sterilization demonstrated favourable results as the structural characteristics of the thin films that were sterilized with UV radiation were not affected by the process. On the contrary, plasma treatment caused changes to the thin films; this renders the outcome of the fabrication and the sterilization of the drug delivery systems less predictable (cracks may lead to early drug release and visual symptoms) and should certainly be considered during further research.

To summarize, future stages of the project may prioritize gravure printing as a coating technique and UV radiation as a sterilization method. These options have shown promising results and may contribute to further advances towards a drug-eluting intraocular lens suitable for commercial production.

Limitations of this study include the relatively small number of specimens and the application of one technique only (AFM) to study the modified IOLs. Further stages of the project could also include a variety of different IOL materials (hydrophilic, hydrophobic, silicone) and types (one-piece, three-piece).

## Conclusions

Spin coating and gravure printing lead to the development of uniform monolayer drug-eluting polymeric thin films on the surface of IOLs, while spray coating has not led to the development of coatings with the desired structural properties. Gravure printing is an industry-oriented technique and is, therefore, more appropriate for further research that may lead to larger-scale production of modified IOLs. UV sterilization does not affect the morphology of the thin films; this technique is, therefore, more likely to prove useful in further stages of this ongoing project, compared to treatment with plasma.
